# Transient receptor potential ankyrin 1 (TRPA1) receptor is involved in chronic arthritis: in vivo study using TRPA1-deficient mice

**DOI:** 10.1186/s13075-015-0904-y

**Published:** 2016-01-08

**Authors:** Ádám Horváth, Valéria Tékus, Melinda Boros, Gábor Pozsgai, Bálint Botz, Éva Borbély, János Szolcsányi, Erika Pintér, Zsuzsanna Helyes

**Affiliations:** Department of Pharmacology and Pharmacotherapy, University of Pécs, Medical School, 12 Szigeti Street, Pécs, 7624 Hungary; János Szentágothai Research Centre, University of Pécs, 20 Ifjúság Street, Pécs, 7624 Hungary; Centre of Neuroscience, University of Pécs, Medical School, Pécs, 20 Ifjúság Street, Pécs, 7624 Hungary; PharmInVivo Ltd., 10 Szondi György Street, Pécs, 7624 Hungary; MTA-PTE NAP B Chronic Pain Research Group, 12 Szigeti Street, Pécs, 7624 Hungary

**Keywords:** TRPA1 receptor-deficient mice, Carrageenan-induced acute inflammation, Adjuvant-induced arthritis, MIA-induced osteoarthritis, In vivo optical imaging, Oedema, Hypersensitivity, Pain

## Abstract

**Background:**

The transient receptor potential ankyrin 1 (TRPA1) is a calcium-permeable cation channel that is expressed on capsaicin-sensitive sensory neurons, endothelial and inflammatory cells. It is activated by a variety of inflammatory mediators, such as methylglyoxal, formaldehyde and hydrogen sulphide. Since only few data are available about the role of TRPA1 in arthritis and related pain, we investigated its involvement in inflammation models of different mechanisms.

**Methods:**

Chronic arthritis was induced by complete Freund’s adjuvant (CFA), knee osteoarthritis by monosodium iodoacetate (MIA) in TRPA1 knockout (KO) mice and C57Bl/6 wildtype mice. For comparison, carrageenan- and CFA-evoked acute paw and knee inflammatory changes were investigated. Thermonociception was determined on a hot plate, cold tolerance in icy water, mechanonociception by aesthesiometry, paw volume by plethysmometry, knee diameter by micrometry, weight distribution with incapacitance tester, neutrophil myeloperoxidase activity and vascular leakage by in vivo optical imaging, and histopathological alterations by semiquantitative scoring.

**Results:**

CFA-induced chronic mechanical hypersensitivity, tibiotarsal joint swelling and histopathological alterations, as well as myeloperoxidase activity in the early phase (day 2), and vascular leakage in the later stage (day 7), were significantly reduced in TRPA1 KO mice. Heat and cold sensitivities did not change in this model. Although in TRPA1 KO animals MIA-evoked knee swelling and histopathological destruction were not altered, hypersensitivity and impaired weight bearing on the osteoarthritic limb were significantly decreased. In contrast, carrageenan- and CFA-induced acute inflammation and pain behaviours were not modified by TRPA1 deletion.

**Conclusions:**

TRPA1 has an important role in chronic arthritis/osteoarthritis and related pain behaviours in the mouse. Therefore, it might be a promising target for novel analgesic/anti-inflammatory drugs.

## Background

Chronic arthritis is one of the greatest health problems worldwide due to its high prevalence and insufficient therapeutic outcomes [1, 2]. Pain is the key complaint for arthritic patients, but its precise mechanism is poorly understood and its treatment is also a great unresolved problem. The currently used analgesics are often ineffective or their long-term use induces severe adverse effects [3–5]. Therefore, there is a pressing need to understand the pathomechanisms of arthritis-related pain and identify more effective pharmacological targets for analgesia. Investigation of the regulatory role and activation mechanisms of peptidergic sensory nerves and the complexity of neuro-immune interactions in this condition can be a promising research area [6–9].

Transient receptor potential ankyrin 1 (TRPA1) is a calcium-permeable non-selective cation channel predominantly expressed on capsaicin-sensitive primary sensory neurons, co-localized with the transient receptor potential vanilloid 1 (TRPV1) receptor in over 90 % of these cells [10, 11]. Besides the nociceptors, functional TRPA1 has also been described on non-neuronal cells, such as keratinocytes [12], fibroblasts [13], synoviocytes [14], macrophages [15, 16], lymphocytes [17], thymocytes [17] and endothelial cells [18] suggesting its complex involvement in inflammatory mechanisms. TRPA1 was characterized originally as a noxious cold- (<17 °C) activated channel [11], although its function in cold sensation is still a matter of debate. Several studies showed that TRPA1 is not required for normal cold sensitivity [19, 20], but others suggest that it acts as a major sensor for noxious cold [20], contributes to cold nociception and cold hypersensitivity after inflammation and nerve injury [21–23]. TRPA1 is also directly stimulated by intracellular calcium [24] and a broad range of noxious endogenous oxidative products, such as 4-hydroxy-2-nonenal, hydrogen peroxide, hypochloride, hydrogen sulphide, 15-delta prostaglandin J2 [25–28]. Furthermore, there are several exogenous irritants like mustard oil (allyl isothiocyanate: AITC) [29], cinnamaldehyde [30, 31], allicin [32, 33] and formalin [34] that are known to be potent agonists of TRPA1. Inflammatory mediators, such as bradykinin and serotonin, can sensitize this receptor and increase the responsiveness of the nerve endings [19, 35]. These findings suggest that TRPA1 may be involved in the development and maintenance of arthritic pain, but the precise mechanisms are still unknown.

Few data are available on the involvement of this receptor in inflammation and pain. Previously, some studies showed the role of TRPA1 in nociceptive processes in vivo using selective antagonists of the receptor. The pharmacological blockade of TRPA1 using intraplantar injection of AP-18 24 hours after complete Freund’s adjuvant (CFA) injection and intraplantar, intraperitoneal or intrathecal administration of HC-030031 1, 7 and 28 days after the administration of the adjuvant significantly attenuated mechanical hypersensitivity in mice and cold hypersensitivity in rats [36, 37]. Oral HC-030031 significantly reversed mechanical hypersensitivity in the CFA model of inflammatory pain at the 24-hour time point and the spinal nerve ligation model of neuropathic pain in rats 6 weeks post surgery [38]. HC-030031 given intraperitoneally or orally significantly reduced formalin- and AITC-evoked nocifensive behaviours [34, 38]. Its intraplantar administration prevented and reversed carrageenan-induced mechanical hypersensitivity in rats [10], inhibited AITC- and carrageenan-induced paw oedema in mice at the 3- and 6-hour time point [39]. In the monosodium iodoacetate (MIA)-induced osteoarthritis (OA) model systemic or intra-articular HC-030031 failed to block weight asymmetry and ongoing pain at the 1-hour time point [40], systemic injection of another TRPA1-antagonist, A-967079, reduced the evoked neuronal responses to high-intensity mechanical stimulation, but did not alter their spontaneous firing in osteoarthritic animals [41].

Although TRPA1-deficient mice are also valuable tools to investigate the role of this receptor in vivo, only few arthritis studies have been performed with these. Two articles reported that TRPA1 knockout (KO) mice did not develop acute pain, thermal or mechanical hypersensitivity after intraplantar injection of bradykinin or AITC, but displayed reduced sensitivity to intense cold and punctate mechanical stimulation [19, 23]. In contrast, one study showed that TRPA1 KO mice exhibited mechanical hypersensitivity 24 hours following CFA injection [36]. In a chronic model of inflammatory pain induced by intra-articular CFA, in TRPA1 KO mice mechanical hypersensitivity developed only 24 hours after CFA administration, but it was significantly smaller than in the wildtypes during the whole 4-week period [42]. TRPA1-deficient mice displayed attenuated carrageenan- and AITC-induced acute inflammatory paw oedema at the 3- and 6-hour time point [39]. The age-dependent role for TRPA1 in pain behaviour occurring in the adjuvant-induced arthritis model has very recently been revealed: old (>18 months old) TRPA1 KO mice developed significantly lower mechanical hypersensitivity as compared to their wildtypes throughout the 8-week experimental period, while the young (3–6 months old) TRPA1-deficient ones revealed that only during the first 2 weeks [43].

There are only few data regarding the role of TRPA1 in inflammation and pain, there are no knockout studies providing evidence for its precise role in chronic inflammatory pain processes in various time points, except for one article [42]. Therefore, in the present study, we aimed to analyze its involvement in chronic arthritis of different mechanisms and related nociception in comparison with acute models using TRPA1-deficient mice.

## Methods

### Ethics statement

All experimental procedures were carried out according to the 1998/XXVIII Act of the Hungarian Parliament on Animal Protection and Consideration Decree of Scientific Procedures of Animal Experiments (243/1988), complied with the recommendations of the International Association for the Study of Pain (IASP). The studies were approved by the Ethics Committee on Animal Research of the University of Pécs (licence number: BA 02/2000–2/2012).

### Experimental animals

Experiments were carried out on male and female TRPA1-deficient mice (TRPA1 KO) and their wildtype (WT) counterparts (8–12 weeks, 20–30 g). Heterozygous TRPA1-deleted mice generated on the C57Bl/6 background were kindly donated by Pierangelo Geppetti (University of Florence, Italy). These mice were generated and characterized as described in detail in earlier publications [19, 23]. They were bred and kept in the Laboratory Animal House of the Department of Pharmacology and Pharmacotherapy of the University of Pécs at 24–25 °C, provided with standard mouse chow and water ad libitum and maintained under a 12-hour light–dark cycle. Each mouse genotype was confirmed by PCR analysis.

### CFA-induced chronic inflammatory pain model

The chronic joint inflammation was induced by intraplantar injection of complete Freund’s adjuvant (CFA, heat-killed Mycobacterium tuberculosis suspended in paraffin oil, 1 mg/ml; Sigma-Aldrich, St. Louis, MO, USA) into the right hindpaw and subcutaneously (s.c.) into the tail root (50–50 μl). An additional s.c. injection was administered on the following day into the tail root (50 μl) in order to potentiate the systemic effects. Measurements were performed throughout the 21-day experimental period. In mice, this injection paradigm induces a chronic arthritis in the tibiotarsal joint on the injection side transiently accompanied by mild systemic symptoms in the early phase, such as weight loss, fever and spontaneous motility decrease. However, arthritic symptoms do not develop in the contralateral hindlimb [44].

### MIA-induced osteoarthritis model

Mice were anaesthetized with intraperitoneally (i.p.) administered ketamine-xylazine (100/10 mg/kg). Osteoarthritis was induced by an injection of 20 μl, 25 mg/ml monosodium iodoacetate (MIA, Sigma-Aldrich, St. Louis, MO, USA) in 0.9 % saline into the left knee joint cavity through the patellar ligament. Measurements were performed throughout the 21-day experimental period [45].

### Carrageenan-induced acute inflammatory pain model

Acute paw inflammation was evoked by intraplantar injection of 3 % carrageenan (dissolved in 0.9 % sodium chloride , Sigma-Aldrich, St. Louis, MO, USA) into the right hindpaw (50 μl). Measurements were performed throughout the 24-hour experimental period [46].

### CFA-induced acute inflammatory pain model

Mice were anaesthetized with i.p. ketamine-xylazine (100/10 mg/kg). For induction of joint inflammation, the knees of mice were injected intra-articularly with 20 μl CFA (Sigma-Aldrich, St. Louis, MO, USA) into the right knee joint and 20 μl saline (0.9 % sodium chloride) into the left knee joint. Measurements were carried out over a period of 24 hours [42].

### Measurement of mechanical hypersensitivity

Mechanical sensitivity of the plantar surface of the paw was measured by dynamic plantar aesthesiometry (DPA, Ugo Basile 37400, Comerio, Italy). Mechanonociceptive thresholds were expressed in gram (g) [44, 47].

### Measurement of paw swelling

Paw volume was measured by plethysmometry (Ugo Basile Plethysmometer 7140, Comerio, Italy), and was expressed in cubic centimeter (cm^3^) [44, 47].

### Measurement of thermal hypersensitivity

The thermonociceptive threshold of the paw was determined on an increasing-temperature hot plate (IITC Life Science, Woodland Hills, CA, USA) heated up from 30 °C at a rate of 12 °C/min until the animal either exhibited nocifensive responses (lifting, shaking or licking either hindpaw) or the maximum value (53 °C) was reached. Hypersensitivity was expressed in °C drop of thermonociceptive threshold compared to the control values [48].

### Measurement of cold sensitivity of the paw

The cold sensitivity was determined by the withdrawal latency after immersing the hindpaw in 0 °C water. It was expressed as withdrawal latency decrease compared to the control values [49].

### Measurement of knee diameter

The anteroposterior and mediolateral diameter of the knee joints were measured with a digital micrometer (Mitutoyo Corporation, Kawasaki, Japan). Knee joint thickness was expressed in millimeter (mm) [42].

### Measurement of spontaneous weight distribution

Spontaneous weight bearing on the two hindlimbs was determined by an incapacitance tester (Linton Instrumentation, Diss, England). The percentage of weight distributed on the ipsilateral hindlimb ([weight on the right hindlimb/(weight on the left + weight on the right)] x 100) was compared before and after the MIA administration [50].

### In vivo bioluminescence imaging of myeloperoxidase

Neutrophil myeloperoxidase (MPO) activity was assessed with luminol-derived bioluminescence. Luminol (5-amino-2,3-dihydro-1,4-phthalazinedione) sodium salt (150 mg/kg, Gold Biotechnology, Olivette, MO, USA) dissolved in sterile phosphate-buffered saline (PBS, 20 mg/ml) was injected intraperitoneally into anaesthetized mice (100/10 mg/kg ketamine-xylazine i.p.) on days 2 and 7 post CFA administration. Bioluminescence imaging was performed 10 minutes post injection using the IVIS Lumina II (PerkinElmer, Waltham, MA, USA; 60s acquisition, f/stop = 1, binning = 8). Identical regions of interest (ROIs) were applied around the ankles and luminescence was expressed as total radiance (total photon flux/s) [51, 52].

### In vivo fluorescence imaging of vascular leakage

Vascular leakage was visualized on indocyanine green (ICG)-based fluorescence imaging. ICG (0.5 mg/kg, Sigma-Aldrich, St. Louis, MO, USA) dissolved in Kolliphor HS 15 (Sigma-Aldrich, St. Louis, MO, USA) was injected intravenously into anaesthetized mice (100/10 mg/kg ketamine-xylazine i.p.) on days 2 and 7 following CFA administration. Fluorescence imaging was performed 20 minutes post injection using the IVIS Lumina II (PerkinElmer, Waltham, MA, USA; auto acquisition time, f/stop = 1, binning = 2, excitation: 745 nm, emission filter: >800 nm). Data were analyzed and ROIs were drawn around the ankle joints. Fluorescence was expressed as total radiant efficiency ([photons/s/cm2/sr]/[μW/cm2]) [51].

### Histology and evaluation of joint inflammation

Animals were terminally anaesthetized using sodium pentobarbital (50 mg/kg, i.p.). They were euthanized 10 days after CFA (the maximum point of the inflammation) and 22 days after MIA administration (the peak of the tissue damage). Ankle and knee joints were excised, fixed in formaldehyde, decalcified, dehydrated, embedded in paraffin, sliced into sections (3–5 μM) and stained with hematoxylin and eosin or Safranin O for detecting collagen depositions and fibroblasts [44, 47]. Arthritic changes were scored by an observer blinded from the study. CFA-induced histopathological changes were scored using a scale of 0 to 3 according to (1) infiltration of mononuclear cells into the areolar tissue, (2) synovial cell lining hyperplasia, and (3) cartilage destruction. The scores for each of the three criteria were accumulated to generate a composite arthritis score ranging between 0 and 9. MIA-induced histological changes were characterized with a modified Mankin semiquantitative scoring system and additional parameters [53–55]. The Mankin score assesses structure (0–6), cellularity (0–4), matrix staining (0–4), and tidemark integrity (0–1). In addition synovial hyperplasia and synovial inflammatory cells infiltration on a scale of 0 to 3 and the presence of osteophyte formation (0–1) were scored. Mean scores were determined from the sections of different animals, and composite score values were calculated from these mean scores.

### Statistical analysis

Results are expressed as mean ± standard error of the mean (SEM). Statistical evaluation was carried out by GraphPad Prism 5 (GraphPad Software, San Diego, CA, USA). Mechanical hypersensitivity, paw oedema, thermal hypersensitivity, cold allodynia, knee joint swelling, spontaneous weight distribution, results from bioluminescence and fluorescence imaging were evaluated by repeated measures two-way analysis of variance (ANOVA) followed by Bonferroni’s multiple comparison test. In the histological study one-way ANOVA followed by Bonferroni’s multiple comparison test were used. In all cases *p* < 0.05 was considered to be significant.

## Results

### CFA-induced mechanical hypersensitivity and paw oedema are attenuated in TRPA1-deficient mice

**Fig. 1:**
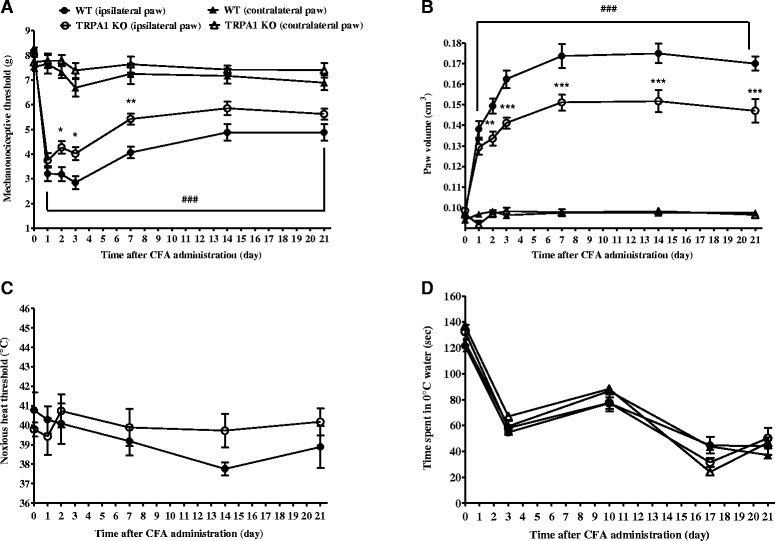
Attenuated CFA-induced chronic mechanical hypersensitivity and oedema in TRPA1 KO mice. CFA-evoked (**a**) mechanical hypersensitivity (n = 14–17/group), (**b**) oedema (n = 14–17/group), (**c**) thermal hypersensitivity (n = 7–8/group), and (**d**) cold sensitivity (n = 7–8/group) of the hindpaw of TRPA1 KO mice as compared to their WT counterparts throughout the 21-day experimental period. Data are shown as means ± SEM, *p < 0.05, **p < 0.01, ***p < 0.001 (vs. WT ipsilateral paw), ^###^ *p* < 0.001 (vs. respective contralateral paw); two-way ANOVA followed by Bonferroni’s multiple comparison test. *ANOVA* analysis of variance, *CFA* complete Freund’s adjuvant, *KO* knockout, *SEM* standard error of the mean, *TRPA1* transient receptor potential akyrin 1, *WT* wildtype

In WT animals an approximate 60 % decrease of the mechanonociceptive thresholds developed 1 day after the adjuvant injection (from 8.19 ± 0.13 g to 3.21 ± 0.29 g), which gradually decreased to 40 % (4.88 ± 0.33 g) by the end of the study. Significantly reduced mechanical hypersensitivity was observed in the TRPA1-deleted group starting on day 2 of the experiment (Fig. 1a). Considerable hindpaw oedema developed in WT mice after the induction of arthritis reaching a maximal swelling of approximately 83 % on day 14 (from 0.09 ± 0.001 cm^3^ to 0.18 ± 0.005 cm^3^). The oedema was significantly smaller in TRPA1 KO animals with a maximum of 54 % (0.15 ± 0.005 cm^3^; Fig. 1b). The thermonociceptive thresholds of the inflamed paws of both WT and TRPA1 KO mice were similar to the baseline values of naive mice (WT: 40.78 ± 0.09 °C vs. TRPA1 KO: 39.79 ± 0.36 °C; Fig. 1c). The time spent in 0 °C water did not differ basically between the TRPA1 KO and WT mice (WT ipsilateral paw: 121.71 ± 4.44 sec vs. TRPA1 KO ipsilateral paw: 132.56 ± 5.46 sec; WT contralateral paw: 124.36 ± 6.37 sec vs. TRPA1 KO contralateral paw: 136.81 ± 4.56 sec). Cold tolerance similarly decreased in all groups during the experimental period independently of the inflammation suggesting hypersensitivity induced by the repeated measurements (Fig. 1d).

### Neutrophil myeloperoxidase activity is reduced in the early phase and vascular leakage is decreased in the late stage of the arthritis in TRPA1 KO animals

**Fig. 2:**
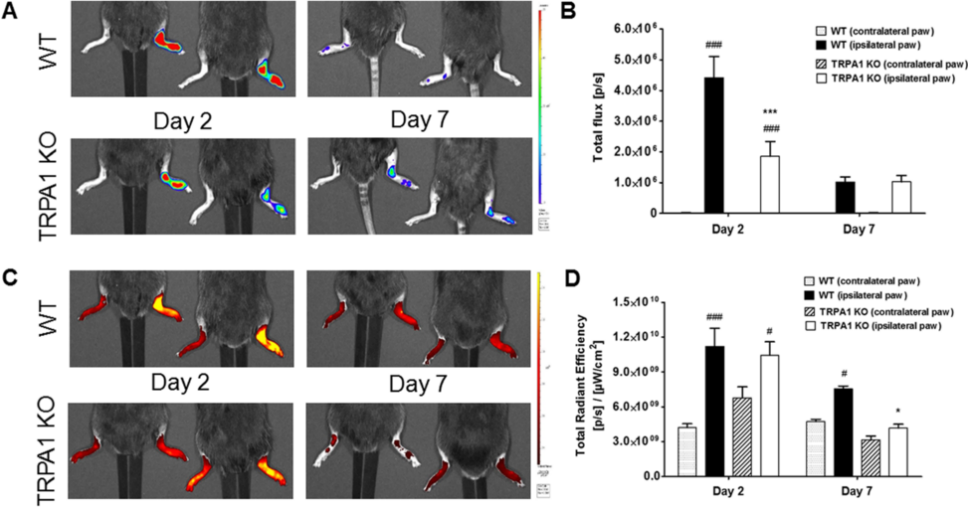
Decreased CFA-evoked neutrophil myeloperoxidase activity and vascular leakage in the ankle joints of TRPA1 KO mice. **a** Representative images of luminol activity showing neutrophil myeloperoxidase activity, and (**b**) quantification of luminescence in the diseased joints on days 2 and 7. **c** Representative images of indocyanine green fluorescence demonstrating plasma protein extravasation in the ipsilateral joints, and (**d**) quantitative analysis of the fluorescence intensity at the same time points. Data are shown as means ± SEM of n = 5–7 mice/group, *p < 0.05, ***p < 0.001 (vs. WT ipsilateral joint), ^#^ *p* < 0.05, ^###^ *p* < 0.001 (vs. respective contralateral joint); two-way ANOVA followed by Bonferroni’s multiple comparison test. *ANOVA* analysis of variance, *CFA* complete Freund’s adjuvant, *KO* knockout, *SEM* standard error of the mean, *TRPA1* transient receptor potential akyrin 1, *WT* wildtype

Luminol-derived bioluminescence revealed an increase in neutrophil-derived MPO activity in the arthritic ankle joints of both groups, being significantly smaller in the KO strain in the early phase (day 2) (Fig. 2a). The fluorescence was similarly high in the ankle joints of both groups in the early phase, demonstrating a remarkable enhancement of plasma extravasation. In the late phase (day 7), plasma extravasation diminished in both groups compared to the early phase, but significant difference was detected in TRPA1 KO mice (Fig. 2b).

### CFA-induced histopathological severity was reduced in the tibiotarsal joint of TRPA1 KO mice

**Fig. 3:**
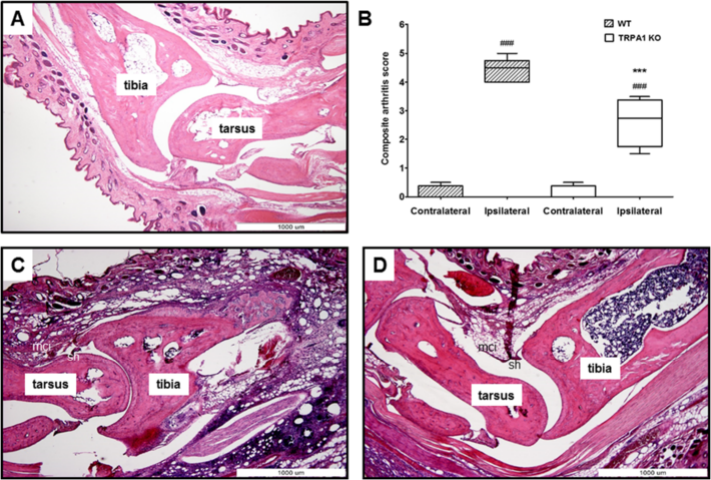
Decreased severity of CFA-induced histopathological alterations in the tibiotarsal joint of TRPA1 KO mice. Representative slides stained with hematoxylin and eosin of (**a**) an intact tibiotarsal joint of a WT, (**c**) arthritic WT, and (**d**) arthritic TRPA1 KO mouse obtained on day 10 (40× magnification; mononuclear cell infiltration (mci), synovial cell lining hyperplasia (sh)). **b** Semiquantitative composite arthritis scores obtained on the basis of synovial cell lining hyperplasia, mononuclear cell infiltration and cartilage destruction. Box plots represent medians of composite scores for n = 4–5 mice/group; ***p < 0.001 (vs. WT ipsilateral joint), ^###^ *p* < 0.001 (vs. respective contralateral joint); one-way ANOVA followed by Bonferroni’s multiple comparison test. *ANOVA* analysis of variance, *CFA* complete Freund’s adjuvant, *KO* knockout, *TRPA1* transient receptor potential akyrin 1, *WT* wildtype

The tibiotarsal joints of WT mice showed remarkably enhanced inflammatory cell infiltration into the areolar tissue, marked synovial cell lining hyperplasia and minimal cartilage destruction (Fig. 3c). In contrast, TRPA1-deficient mice showed reduced infiltration of inflammatory cells into the areolar tissue and moderate hyperplasia of the synovial cell lining, but cartilage damage was not detected (Fig. 3d). Semiquantitative scoring of composite arthritic changes in CFA-injected tibiotarsal joints found the severity of arthritis was significantly decreased in KO animals (WT ipsilateral joint: 4.4 ± 0.19 vs. TRPA1 KO ipsilateral joint: 2.63 ± 0.43) (Fig. 3b).

### MIA-induced pain behaviour was decreased in TRPA1-deleted mice

**Fig. 4:**
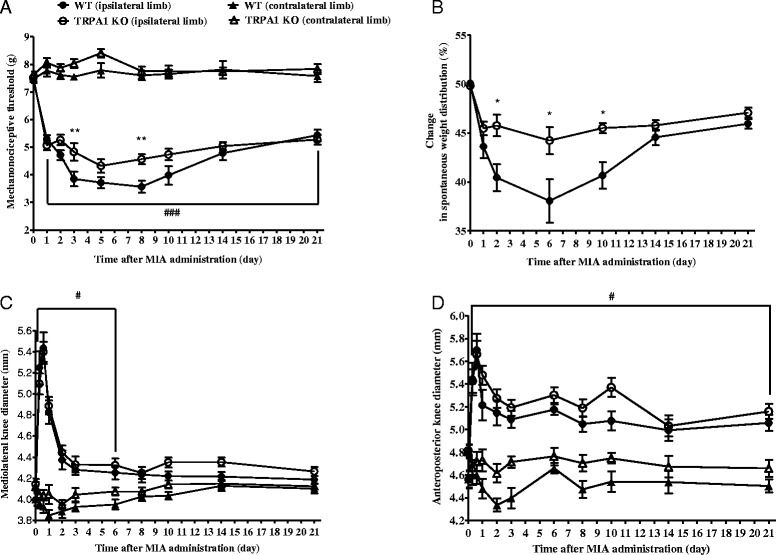
Decreased MIA-induced pain behaviours in TRPA1 KO mice. MIA-evoked (**a**) mechanical hypersensitivity, (**b**) percentage of spontaneous weight distribution and (**c**-**d**) mediolateral and anteroposterior diameters of the ipsilateral knee joints of TRPA1 KO mice as compared to their WT counterparts throughout the 21-day experimental period. Results are shown as means ± SEM of n = 10–15 mice/group, *p < 0.05 (vs. WT ipsilateral limb), ^#^ *p* < 0.05, ^###^ *p* < 0.001 (vs. respective contralateral limb), two-way ANOVA followed by Bonferroni’s multiple comparison test. *ANOVA* analysis of variance, *KO* knockout, *MIA* monosodium iodoacetate, *SEM* standard error of the mean, *TRPA1* transient receptor potential akyrin 1, *WT* wildtype

The basal mechanonociceptive thresholds were 7.42 ± 0.1 g and 7.51 ± 0.13 g in the WT and TRPA1 KO groups, respectively. MIA injection induced a 27–52 % drop of the mechanonociceptive threshold in WT mice and 30–43 % in the KO animals. However, reduced hypersensitivity was measured in the TRPA1 KO group from the 3^rd^ to the 11^th^ day, the difference between the two groups was significant on the 3^rd^ and 8^th^ days of the study (Fig. 4a).

MIA-induced reduction in weight bearing of the ipsilateral limb indicates the development of spontaneous pain. Significantly less decrease of weight distribution was observed in TRPA1 KO mice on the 2^nd^, 6^th^ and 10^th^ days compared to their WTcontrols (Fig. 4b).

MIA evoked a remarkable oedema of the knee joint in both WT and TRPA1 KO mice. The maximal swelling was detected 6 hours after the injection measured mediolaterally (WT: from 3.99 ± 0.06 mm to 5.34 ± 0.151 mm, TRPA1 KO: from 4.15 ± 0.05 mm to 5.40 ± 0.09 mm). Then swelling decreased gradually from the 2^nd^ day in both groups (Fig. 4c-d).

### Histopathological severity did not differ between the MIA-injected knees of TRPA1 KO and WT mice

**Fig. 5:**
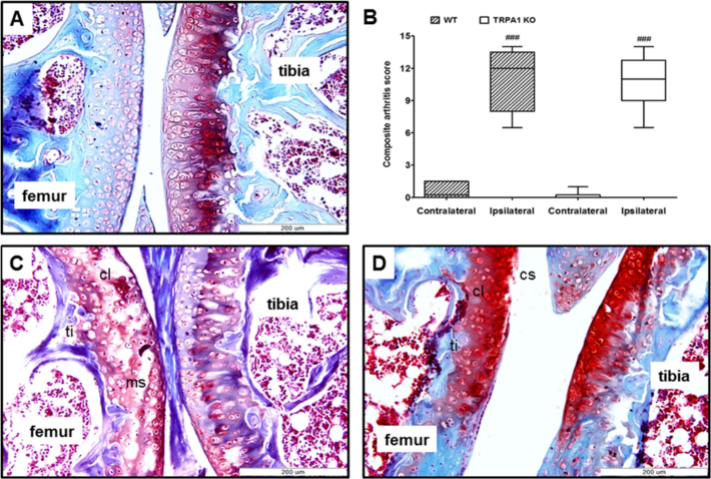
Similar histopathological severity of the MIA-injected knees of TRPA1 KO and WT mice. Representative slides stained with Safranin O of (**a**) an intact knee joint, (**c**) osteoarthritic WT, and (**d**) TRPA1 KO mouse knee obtained on day 22 (200× magnification; roughened cartilage surface (*cs*), moderate disorganization and cell loss (*cl*), reduced matrix staining (*ms*), disrupted tidemark integrity (*ti*)). **b** Modified Mankin semiquantitative scores obtained on the basis of structure, cellularity, matrix staining, tidemark integrity, synovial hyperplasia, synovial inflammatory cells infiltration, and osteophyte formation. Box plots represent medians of composite scores for n = 6–9 mice/group; ^###^ *p* < 0.001 (vs. respective contralateral joint), one-way ANOVA followed by Bonferroni’s multiple comparison test. *ANOVA* analysis of variance, *KO* knockout, *MIA* monosodium iodoacetate, *TRPA1* transient receptor potential akyrin 1, *WT* wildtype

The MIA-injected knee joints of both WT and TRPA1 KO mice showed similar pictures: roughened cartilage surface, moderate disorganization and cell loss, reduced matrix staining, often disrupted tidemark integrity (Fig. 5a-d).

### Similar mechanical hypersensitivity, paw oedema, thermal hypersensitivity and cold sensitivity were detected in WT and TRPA1 KO mice after carrageenan administration

**Fig. 6:**
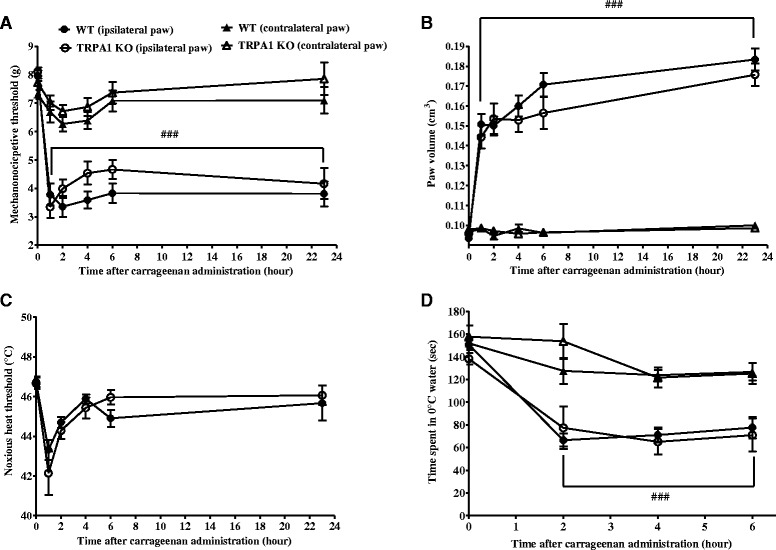
No change of acute carrageenan-evoked hypersensitivity and paw oedema in TRPA1 KO mice. Carrageenan-induced (**a**) mechanical hypersensitivity (n = 12–13/group), (**b**) oedema (n = 12–13/group), (**c**) thermal hypersensitivity (n = 12–13/group), and (**d**) cold sensitivity (n = 7–8/group) of the hindpaw of TRPA1 KO mice as compared to their WT counterparts throughout the 24-hour experimental period. Data are shown as means ± SEM, ^###^ *p* < 0.001 (vs. respective contralateral paw); two-way ANOVA followed by Bonferroni’s multiple comparison test. *ANOVA* analysis of variance, *KO* knockout, *MIA* monosodium iodoacetate, *SEM* standard error of the mean, *TRPA1* transient receptor potential akyrin 1, *WT* wildtype

Similar mechanical hypersensitivity developed in the carrageenan-injected paws both in the WT and TRPA1 KO groups 1 hour after the injection, the mechanonociceptive threshold decreased from 7.96 ± 0.19 g to 3.78 ± 0.38 g and 8.11 ± 0.15 g to 3.35 ± 0.39 g, respectively (Fig. 6a).

An approximate 60 % paw oedema developed 1 hour after the carrageenan administration in both groups (WT: from 0.09 ± 0.001 cm^3^ to 0.15 ± 0.005 cm^3^, TRPA1 KO: from 0.09 ± 0.001 cm^3^ to 0.14 ± 0.005 cm^3^), reached the maximum of 85–95 % at the 23-hour time point (Fig. 6b).

The noxious heat threshold decreased by the carrageenan administration after 1 hour in both groups, then returned to the control values (WT: 46.74 ± 0.28 °C vs. TRPA1 KO: 46.66 ± 0.29 °C, Fig. 6c). The time spent in 0 °C water decreased to 50 % compared to the initial values in both groups (Fig. 6d).

### Mechanical hypersensitivity and oedema similarly developed in CFA-induced acute knee joint inflammation of TRPA1 KO and WT mice

**Fig. 7:**
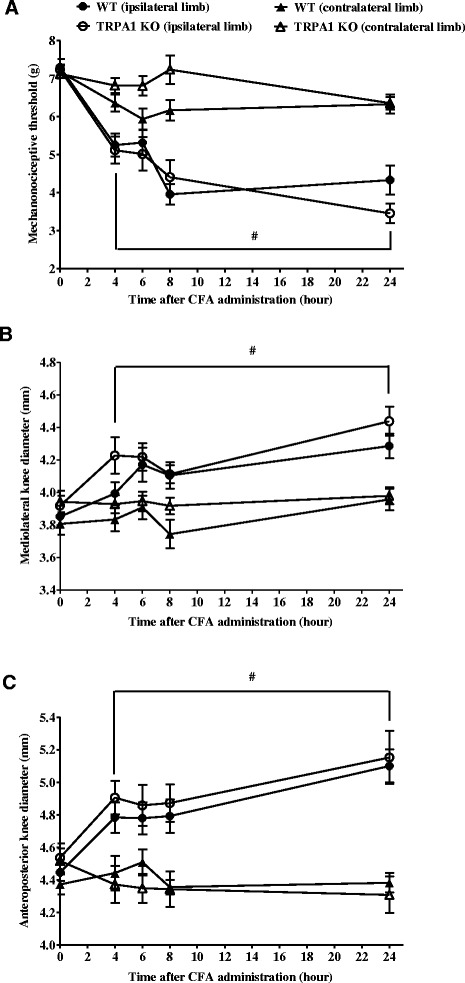
CFA-induced acute knee joint pain and oedema are not influenced by TRPA1 deletion. CFA-evoked acute (**a**) mechanical hypersensitivity, (**b**-**c**) mediolateral and anteroposterior diameters of the ipsilateral knee joints of TRPA1 KO mice as compared to their WT counterparts throughout the 24-hour experimental period. Results are shown as means ± SEM of n = 11–12 mice/group, ^#^ *p* < 0.05 (vs. respective contralateral limb), two-way ANOVA followed by Bonferroni’s multiple comparison test. *ANOVA* analysis of variance, *CFA* complete Freund’s adjuvant, *KO* knockout, *SEM* standard error of the mean, *TRPA1* transient receptor potential akyrin 1, *WT* wildtype

Four hours after CFA injection, the mechanonociceptive thresholds of the ipsilateral limbs decreased similarly by 30 % (WT: from 7.30 ± 0.22 g to 5.25 ± 0.30 g, TRPA1 KO: from 7.24 ± 0.13 g to 5.12 ± 0.35 g), and reached its maximum of 40–50 % at the 8-hour time point remaining stable for the total 24-hour duration of the study (Fig. 7a). CFA induced remarkable swelling of the knee joints in both groups with no significant difference. The mediolateral and anteroposterior diameters increased gradually by 10–15 % at the 24-hour time point (mediolateral: WT: from 3.85 ± 0.02 mm to 4.29 ± 0.08 mm, TRPA1 KO: from 3.92 ± 0.061 mm to 4.44 ± 0.09 mm; anteroposterior: WT: from 4.45 ± 0.05 mm to 5.10 ± 0.10 mm vs. KO: from 4.54 ± 0.09 mm to 5.15 ± 0.16 mm) (Fig. 7b-c).

## Discussion

These results showed that chronic arthritis/osteoarthritis and related pain behaviours are mediated by the TRPA1 receptor activation. Adjuvant-induced oedema and inflammatory mechanical hypersensitivity, as well as MIA-evoked degenerative mechanical hypersensitivity with a potential neuropathic component and reduced weight bearing are diminished in TRPA1 KO mice. Furthermore, we presented the first evidence that TRPA1 is involved in the early neutrophil activation and late plasma extravasation in the CFA arthritis model. In contrast to some data indicating that TRPA1 mediates carrageenan-induced acute mechanical hypersensitivity in rats [10] and paw oedema in mice [39], our results clearly demonstrated that this receptor does not have a pivotal role in acute inflammation and hypersensitivity either in the knee joint or in the paw (Table 1).

**Table 1:**
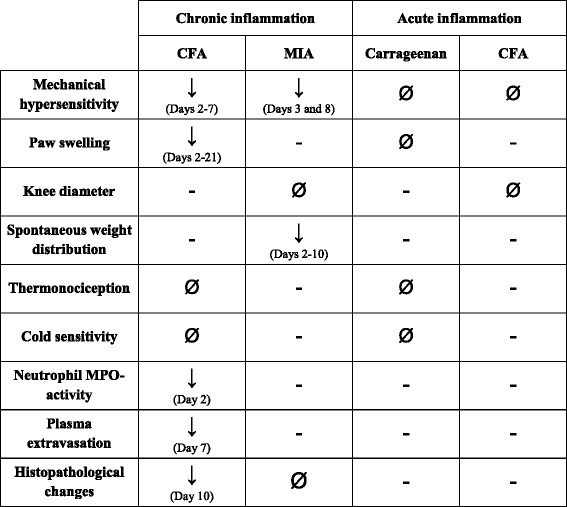
Summary of functional and morphological alterations in different inflammation models in TRPA1 KO mice as compared to their WTs

In TRPA1 KO mice, CFA-induced chronic mechanical hypersensitivity, paw swelling, neutrophil MPO-activation, plasma extravasation and histopathological changes, as well as MIA-evoked mechanical hypersensitivity and spontaneous weight distribution were significantly reduced. In contrast, thermal hypersensitivity and cold sensitivity in the chronic CFA-model, knee diameter, histopathological changes in the MIA-model, and mechanical and thermal hypersensitivity, oedema and cold sensitivity in the carrageenan- and CFA-induced acute models did not significantly differ between the WT and KO groups

*CFA* complete Freund’s adjuvant, *MIA* monosodium iodoacetate, *MPO* myeloperoxidase *TRPA1* transient receptor potential akyrin 1, *KO* knockout, *WT* wildtype

The CFA-, MIA- and carrageenan-induced models are widely used and well-characterized rodent models of acute and chronic inflammation [56–60]. CFA is heat-killed Mycobacterium tuberculosis, muramyl dipeptide involved in the cell wall causes a Th1- and cytokine-driven joint inflammation in rodents, which can mimic the main pathologic features of human rheumatoid arthritis [61–64]. It can also be used to induce acute (1–3 days) and chronic inflammation (3 weeks). In our chronic model TRPA1 KO mice showed significantly decreased mechanical hypersensitivity from days 2 to 7, attenuated oedema from the second day during the total 3-week period, and reduced arthritic changes in the tibiotarsal joint at the 10-day time point as compared to their WT counterparts. Furthermore, we provided the first in vivo bioluminescence and fluorescence imaging data that TRPA1 activation mediates early neutrophil activation (on day 2) and late plasma protein extravasation (on day 7) in chronic arthritis. The regulatory role of TRPA1 receptors in chronic arthritic pain is supported by the finding that TRPA1 deletion hampered ongoing nociception in CFA-induced monoarthritis [42]. However, this study found no difference in knee joint swelling or histology, and concluded that the hyperalgesic function of TRPA1 was dissociated from joint swelling and inflammation [42]. These differences can be explained by the distinct features of these models (intra-articular vs. intraplantar/tail root administration, localized monoarthritis in the knee joint vs. small joints with systemic symptoms, different kinetics) and different investigational techniques (digital micrometer for the knee vs. plethysmometer for the paw). The knockout studies were confirmed by experiments with TRPA1-selective antagonists. Intraperitoneal, intraplantar or intrathecal injection of HC-030031 significantly reduced the long-lasting mechanical hypersensitivity at the 1-, 7- and 28-day time points [37]. The co-expression and interaction of TRPA1 and TRPV1 in a subpopulation of peptidergic, afferent Aδ and C fibres is well known [11, 29, 65, 66]. We previously showed decreased mechanical hypersensitivity, paw swelling and histopathological changes in TRPV1 KO mice in the same CFA model [44]. These results suggest that both TRPA1 and TRPV1 have relevant regulatory roles in long-term inflammation and hypersensitivity. Thermal hypersensitivity did not develop in our CFA model, the noxious heat threshold did not significantly change in response to the inflammatory reaction. Data showing no difference in thermosensitivity of TRPA1 KO and WT mice are in agreement with previous reports suggesting that TRPA1 is not a heat sensor [19, 23, 38]. Meanwhile, cold sensitivity increased in all groups independently of the inflammation suggesting the induction of cold hypersensitivity by the repeated measurements. Therefore, this investigational technique is not suitable for testing nociception in this model. However, pharmacological blockade of TRPA1 abolished CFA-evoked nocifensive behaviour to cold stimulus determined by a distinct methodology (1 % tetrafluoroethane spray on the hindpaw) in mice [37]. Therefore, the in vivo functional relevance of the noxious cold activation of TRPA1 in the processing of different pain modalities still remains an open question.

Intra-articular injection of MIA inhibits a key enzyme activity of glycolysis (glyceraldehye-3-phosphate dehydrogenase) in chondrocytes leading to progressive loss of these cells. The destruction of the articular cartilage, transient synovial inflammation and pain behaviour are similar to the human OA [67–71]. MIA results in decreased weight bearing on the injured limb, movement-evoked pain and hypersensitivity [72, 73]. Previous data showed that chronic joint pain originates from the periphery by the sensitization of primary afferent nerves [74–77]. It is well-known, that TRPA1 receptors are highly expressed on these fibres, but whether these receptors have a role in OA was investigated only with a selective receptor antagonist [40, 41]. Since systemic or intra-articular HC-030031 failed to block pain-related behaviours 1 hour post injection [40], and the blockade of TRPA1 did not reduce the MIA-induced spontaneous firing of sensory neurons [41], it could be concluded that MIA-induced ongoing pain is independent of TRPA1. Our present results with knockout mice support this concept regarding the knee joint swelling and histopathological alterations. However, we found that TRPA1 contributes to MIA-evoked decreased weight bearing and tactile hypersensitivity between days 3 and 10 of the experimental period.

Intraplantar or intra-articular injection of carrageenan is an appropriate acute technique for testing anti-inflammatory drugs [56]. It involves both neurogenic and non-neurogenic mechanisms characterized by prostaglandin production, cyclooxygenase (COX)-2 upregulation, formation of reactive nitrogen and oxygen species, as well as cytokines and other inflammatory mediators [78–82]. In the present study we showed that TRPA1 does not have a role in acute carrageenan-induced paw oedema, mechanical and thermal hypersensitivity. In contrast, previous reports demonstrated that the selective TRPA1-antagonist HC-030031 and/or genetic deletion of TRPA1 inhibited the development and maintenance of carrageenan-induced inflammatory hypersensitivity in rats [10], and paw oedema in mice at the 3- and 6-hour time points [39], respectively. Distinct species, measuring time points, volumes, concentrations, and investigational techniques might be possible explanations for the differences. Similarly to what we found in TRPA1 KO mice, we previously described no difference in the carrageenan model in TRPV1-deficient mice [46].

Similarly to the carrageenan model, acute intra-articular CFA-evoked mechanical hypersensitivity and swelling were not altered by the deletion of the TRPA1 receptor over a 24-hour period, which is in agreement with previous reports [36, 42].

The distinct functional outcomes between the roles of the TRPA1 receptor in our four models can be explained by the wide distribution of TRPA1 on sensory nerves and non-neuronal cells, such as keratinocytes [12], fibroblasts [13], synoviocytes [14], macrophages [15, 16], lymphocytes [17], thymocytes [17] and endothelial cells [18]. Keratinocytes stimulated by TRPA1 agonist have been shown to increase the expression and release of pro-inflammatory interleukins [12], which can activate or sensitize sensory nerve endings [83]. The exposure of thymocytes to cinnamaldehyde accelerated T cell differentiation [17], which has a crucial role in the pathomechanism of CFA-induced arthritis. Macrophages, the other key players of CFA-evoked joint inflammation, also express TRPA1 mediating anti-inflammatory effects [15, 16]. Furthermore, there is a broad range of endogenous TRPA1 agonists produced locally during inflammatory processes that might differently modulate the receptor on the sensory nerves and non-neural structures. This would consequently trigger and/or inhibit the inflammatory cascades.

## Conclusions

Our findings demonstrate an important regulatory role of the TRPA1 receptor in chronic arthritis/osteoarthritis and related pain behaviours in the mouse. Therefore, it might be a promising target for novel analgesic/anti-inflammatory drugs.

## Abbreviations

AITC: Allyl isothiocyanate
ANOVA: Analysis of variance
CFA: Complete Freund’s adjuvant
COX: Cyclooxygenase
DPA: Dynamic plantar aesthesiometer
IASP: International Association for the Study of Pain
ICG: Indocyanine green
i.p.: Intraperitoneal
KO: Knockout
MIA: Monosodium iodoacetate
MPO: Myeloperoxidase
OA: Osteoarthritis
PBS: Phosphate-buffered saline
ROI: Regions of interests
s.c.: Subcutaneous
TRPA1: Transient receptor potential akyrin 1
TRPV1: Transient receptor potential vanilloid 1
WT: Wildtype
